# Addressing infodemic for pandemic preparedness in the digital age: a focus on Middle Africa

**DOI:** 10.3389/fpubh.2024.1275702

**Published:** 2024-09-24

**Authors:** Marthe Bogne Penka, Andrew Tangang, Ernest Alang Wung, Mark Tata Kelese, Patrick Okwen

**Affiliations:** ^1^Effective Basic Services (eBASE) Africa, Yaoundé, Cameroon; ^2^Department of Public Administration and Policy (DPAP), Institute of Local Government Studies (INLOGOV), University of Birmingham, Birmingham, United Kingdom; ^3^Effective Basic Services (eBASE) Africa, Bamenda, Cameroon; ^4^Department of Economic Policy Analysis, Faculty of Economics and Management, University of Dschang, Dschang, Cameroon; ^5^Department of Economics, The University of Bamenda, Bambili, Cameroon

**Keywords:** pandemic, preparedness, infodemic, social media, Africa

## Abstract

**Background:**

The 21st century has brought about a damaging information crisis, significantly challenging and undermining efforts to increase the uptake of scientific research evidence in both policy and practice. The World Health Organization (WHO) recognizes misinformation and disinformation as major drivers of pandemic spread and impact, dedicating a policy brief to pandemic preparedness on this issue. In this study, we examine the impact of mis/disinformation on the use of research evidence in public policy decision-making in West and Central Africa and reflect on how this can inform future pandemic preparedness.

**Objectives:**

What factors affect the uptake of scientific evidence during disease outbreaks in Africa?

**Methods:**

We used the JBI Scoping Review and Prevalence/Incidence Review methodologies to synthesize the best available evidence. A DELPHI survey was conducted in two stages: the first gathered experiences from policymakers, practitioners, and citizens in Cameroon, Nigeria, and Senegal regarding mis/disinformation and its impact. The second stage explored potential situations related to the issues identified in the first stage. Qualitative data analysis was conducted using MAXQDA.

**Results:**

The research identified the origins (*n* = 5), transmission platforms (*n* = 15), cases (*n* = 4), mitigation strategies (*n* = 6), and impacts (*n* = 4) of infodemic on policy design, implementation, and uptake. Online platforms were identified as the main source of infodemic in 53.3% of cases, compared to 46.7% attributed to offline platforms. We conclude that the severity of COVID-19 as a global pandemic has highlighted the dangers of mis/disinformation, with a considerable number of studies from Middle Africa demonstrating a significant negative impact on the uptake of health policies and to an extend evidence informed policy making. It is also imperative to consider addressing evidence hesitancy in citizens through innovative and indigenous approaches like storytelling.

**Discussions:**

Digital technologies, especially social media, play a key role in the propagation of infodemics. For future pandemic preparedness, stakeholders must consider using digital tools and platforms to prevent and mitigate pandemics. This study adds new evidence to the existing body of evidence, emphasizing the need to address infodemics within the context of future pandemic preparedness in Middle Africa.

## Introduction

### Background

Coronavirus disease (COVID-19) is an infectious illness caused by the severe acute respiratory syndrome coronavirus (SARS-CoV-2) (1). It was officially declared a global pandemic on 11 March 2020 (2). According to a UNESCO report from 2020, as of June 2020, Africa had reported 168,592 confirmed cases and 4,758 deaths. Although these numbers are relatively low compared to other regions, there is a great concern about the potential for a high number of unreported cases (3).

The impact of COVID-19 in Africa extends beyond infection rates and mortality to include severe socioeconomic consequences (4). The pandemic has exacerbated existing challenges in Africa, such as gender inequalities, sexual and gender-based violence, job losses in both formal and informal sectors, school dropouts, and food insecurity. These issues have significantly undermined efforts to achieve sustainable development goals (1, 2, 3, 4, 5, 8, 10) (5, 6). While COVID-19 has had a global impact, Sub-Saharan Africa has faced similar health threats in the past, such as Ebola, cholera, and Lassa fever, which have also had comparable effects (7).

The 21st century has ushered in a damaging information crisis that challenges and undermines efforts to increase the uptake of scientific research evidence in both policy and practice (8, 9). While significant progress has been made by scientists and international health regulatory bodies in addressing the global pandemic through the development of vaccines and preventive and protective measures, a single moment of misconception—amplified by social media—can quickly render these efforts futile. This highlights the huge influence of social media during a pandemic (10–12).

This information crisis manifests itself in various forms, including misinformation, disinformation, fake news, and an infodemic. Misinformation refers to false information spread regardless of the intention to mislead (13). On the other hand, disinformation is the deliberate spread of misleading or false information (14). Fake news is purposely fabricated information that mimics the format of mainstream news (15). An infodemic is characterized by an overflow of information, including false or misleading information, circulating in digital and physical environments during a disease outbreak (16).

Infodemics significantly impact citizens' decision-making, often leading to harmful health outcomes and fostering mistrust in healthcare institutions and governments (17). This mistrust can prolong or intensify a pandemic due to the reduced uptake of protective measures. This problem can be further exacerbated by digitalization, where vast amounts of both accurate and false information spread rapidly. In the digital age, the widespread dissemination of false information creates a global situation where vulnerable populations are easily influenced by the overwhelming amount of online content, which in turn affects public policy decisions and the adoption of public health measures (18).

The COVID-19 pandemic has exposed and intensified these challenges, revealing weaknesses in the system for developing policy guidance and best practices (19). According to a 2020 WHO report, in the first 3 months of 2020 alone, nearly 6,000 people worldwide were hospitalized due to coronavirus-related misinformation. Tragically, at least 800 people died as a result of this misinformation, and 60 people suffered complete blindness after drinking methanol, mistakenly believing it was a cure for COVID-19 (20).

The COVID-19 pandemic has exposed the severe impact of infodemics in an era dominated by digital citizens. It has also highlighted and exacerbated the existing weaknesses in the system for developing effective policy guidance and best practices (19).

## Methods

### Sampling: data and countries

We used two systematic review approaches: the scoping review and the prevalence/incidence review, both following the Joanna Briggs Institute guidelines (21). Our searches included a range of electronic databases, such as IDRC, WHO, PubMed, 3ie, Google Scholar, Cochrane, Taylor & Francis, ProQuest, Campbell Collaboration, EBSCO Host, and African Journals, as well as gray literature. Abstracts and full texts were independently screened for eligibility by two reviewers, and two independent reviewers also conducted data extraction. The systematic review focused exclusively on studies conducted in West and Central Africa.

Our Delphi survey was conducted in three countries within these regions—Cameroon, Nigeria, and Senegal—selected based on convenience. The survey was carried out in two stages. The first stage aimed to gather the experiences of stakeholders to identify sources of COVID-19 information, barriers to the adoption of COVID-19 preventive and protective measures, and factors contributing to successful outcomes. The second stage focused on identifying mitigation strategies to reduce the impact of misinformation and disinformation on the use of research evidence during the pandemic.

Given the unique challenges posed by COVID-19, we adopted a specific approach to collect data on misinformation, utilizing Twitter and WhatsApp to gather information from friends and contacts in Cameroon, with a particular focus on COVID-19-related misinformation.

Qualitative data were analyzed using MAXQDA (22).

### Data collection and synthesis

#### Systematic review

We conducted both a scoping review and a prevalence/incidence review.

##### Objectives

- The scoping review aimed to map the landscape of COVID-19 misinformation and disinformation affecting the use of research evidence in public policy decision-making in West and Central Africa.
- The prevalence/incidence review focused on identifying specific cases of COVID-19 misinformation and disinformation affecting the use of research evidence in public policy decision-making.

##### Review questions

- What is the landscape of COVID-19 mis/disinformation impact on the use of research evidence in public policy decisions? (Scoping Review).
- What are the specific cases of COVID-19 misinformation and disinformation affecting decision-making in West and Central Africa? (Prevalence/ Incidence Review).
- What is the prevalence and incidence of COVID-19 misinformation and disinformation among policymakers and citizens in West and Central Africa? (Prevalence/Incidence Review).

##### Search strategy

Condition—Cases of COVID-19 misinformation/disinformation.

Context—West and Central Africa.

Population—Policy makers, Practitioners, Citizens (see Table 10).

##### Searched database

- IDRC
- WHO
- PubMed
- 3ie
- Google Scholar
- Cochrane
- Taylor and Francis
- ProQuest
- Campbell Collaboration
- EBSCO Host
- African Journals Online

##### Exclusion criteria

We excluded all cases of misinformation:

- Not reporting from a West and Central African country.
- Not reporting on the COVID-19 pandemic, education of the girl child, climate change and food security, mother and child health, women empowerment, and advocacy against early marriage and sexual violence.

Two independent authors screened studies and extracted data, with one senior researcher supervising the whole process.

Searches were conducted using Boolean operators developed from the above research questions. Sample from:

**ProQuest** noft(Cases) AND noft(misinformation OR disinformation OR “false information” OR rumor OR “fake news” OR “misleading information” OR deception OR propaganda) AND noft(“West and Central Africa” OR “Sub-Saharan Africa” OR Africa OR “African countries” OR “Developing countries” OR “Low-and middle-income countries”) AND noft(“Policy makers” OR Practitioner^*^ OR Citizen^*^ OR “Decision-makers” OR Politicians OR “Community members” OR Society OR Public OR inhabitant).

#### Delphi survey

##### Objective

The objective of this survey was to gather the stakeholders' experiences and identify sources of information barriers and challenges affecting research evidence in decision-making and determining success. The survey was conducted in two stages:

Stage 1: In this stage, we gathered information on experiences (barriers, determinants of success, and coping strategies) through informal interviews (KIIs). Identified stakeholders were contacted via email by the research team, supported by IDRC. However, the response rate was low, with only nine out of 40 stakeholders agreeing to participate. Data collection was conducted using peer-reviewed and pre-piloted discussion guides on Zoom. The sessions were recorded, transcribed, and coded using Microsoft Word. We extracted data on Microsoft Excel and Maxqda. The insights gained from these interviews were used to identify emerging themes and develop mitigation strategies.

Stage 2: We identified emerging themes and strategies to mitigate the impact of misinformation and disinformation on the use of research evidence in public policy decision-making.

##### Questions

- What are the experiences of stakeholders on the use of COVID-19 preventive and protective measures?
- What are the sources of COVID-19 information?
- What are the barriers affecting the use of COVID-19 preventive and protective measures? What are the determinants of success in facilitating the use of COVID-19 preventive and protective measures?
- What are some mitigation strategies to limit the impact of mis/disinformation on the use of COVID-19 preventive and protective measures?

## Results

### Evidence synthesis

**Figure 1:**
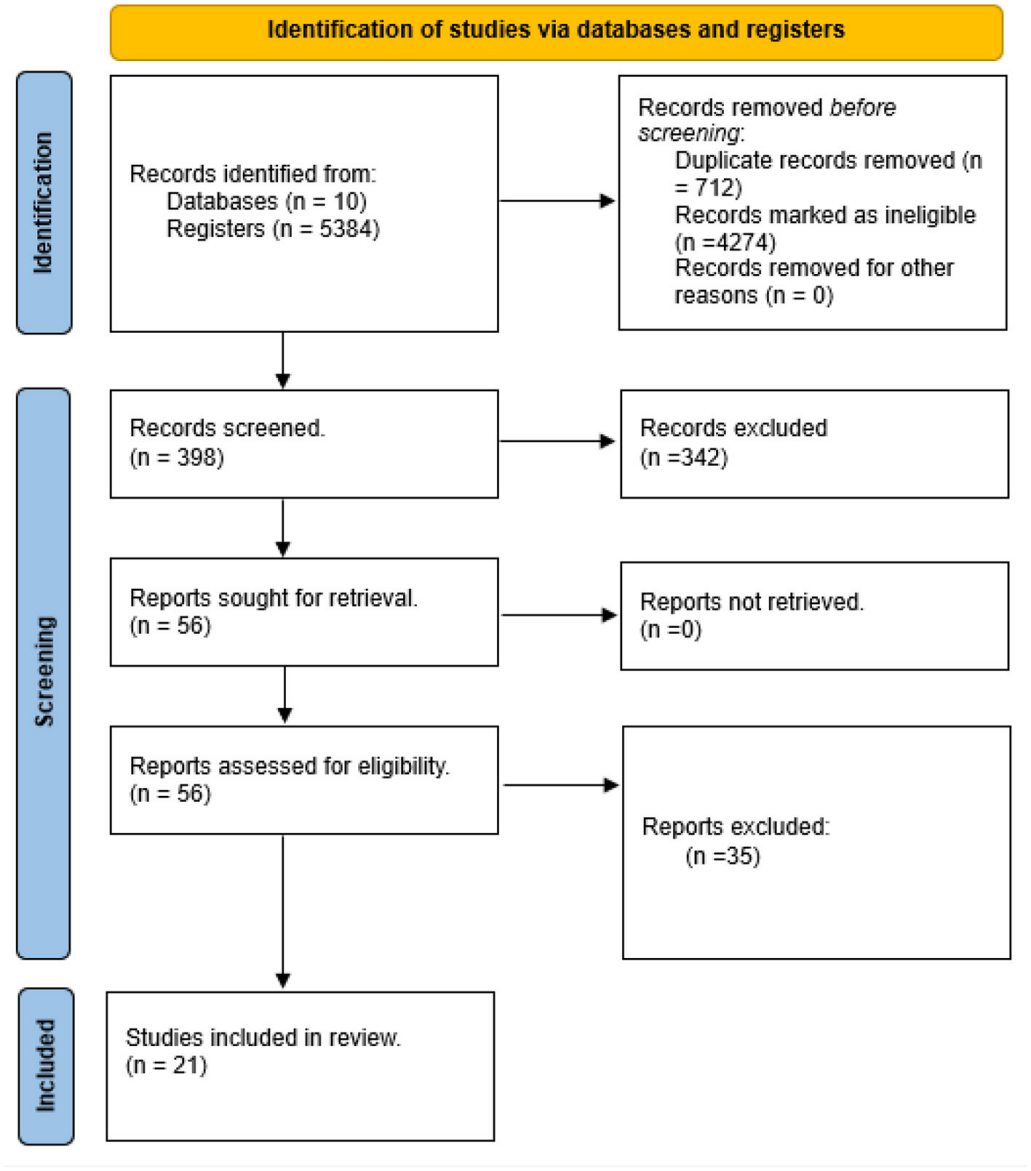
PRISMA flow diagram: visualization of the process involving identification of records from databases, screening of records, assessing reports for eligibility, inclusion of eligible studies and exclusion of non-eligible reports with reasons for exclusion.

A total of 398 studies were identified for screening after the removal of duplicates. Of these, 342 studies were excluded based on the inclusion/exclusion criteria. Out of 56 articles retrieved, 21 studies were deemed eligible. Studies were reported from nine out of 23 countries in West and Central Africa (see Figure 1).

#### Transmission mechanism of mis/disinformation

**Figure 2:**
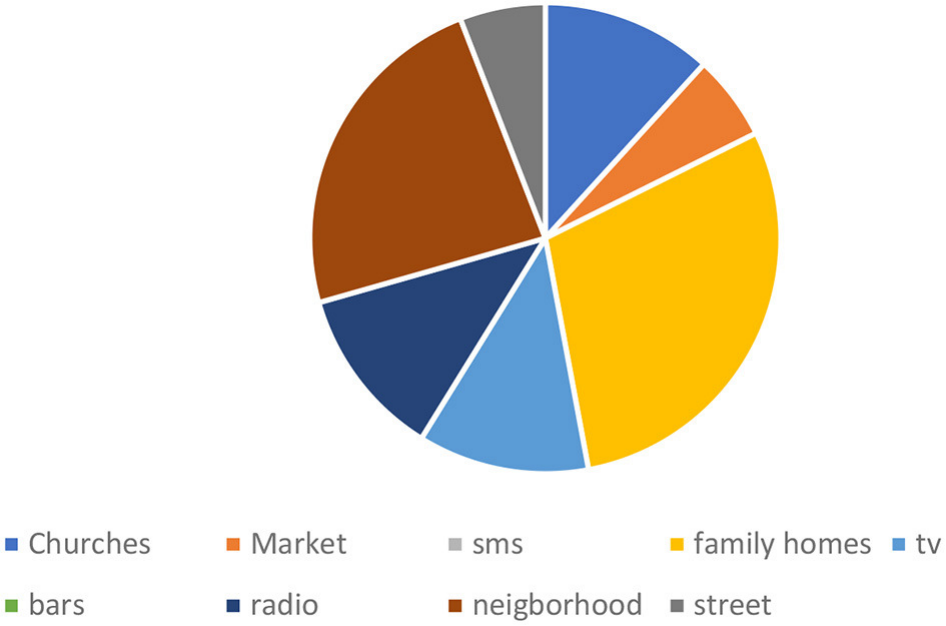
Offline sources of mis/disinformation.

**Figure 3:**
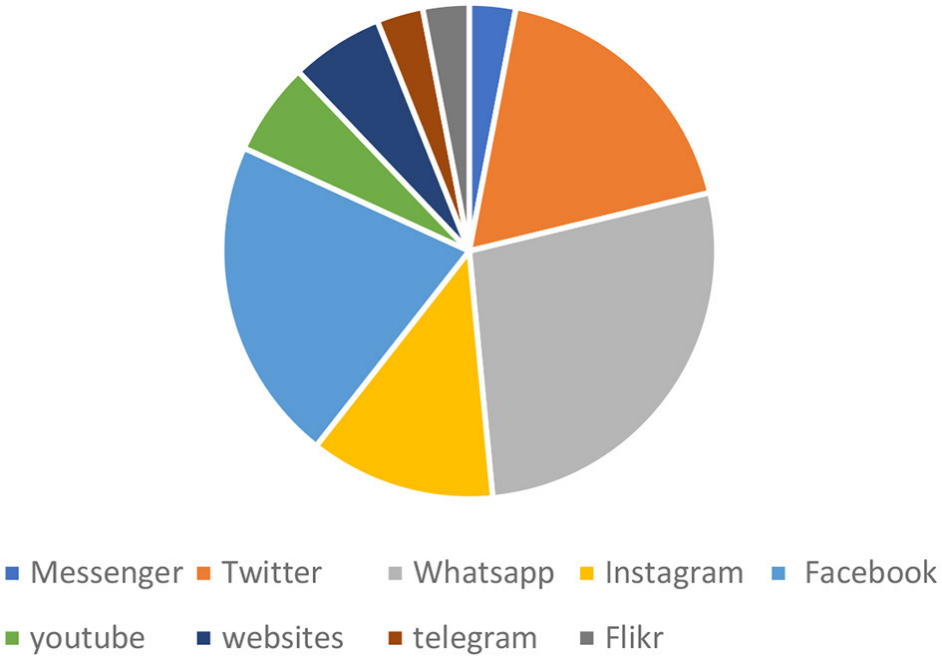
Online sources of mis/disinformation.

**Figure 4:**
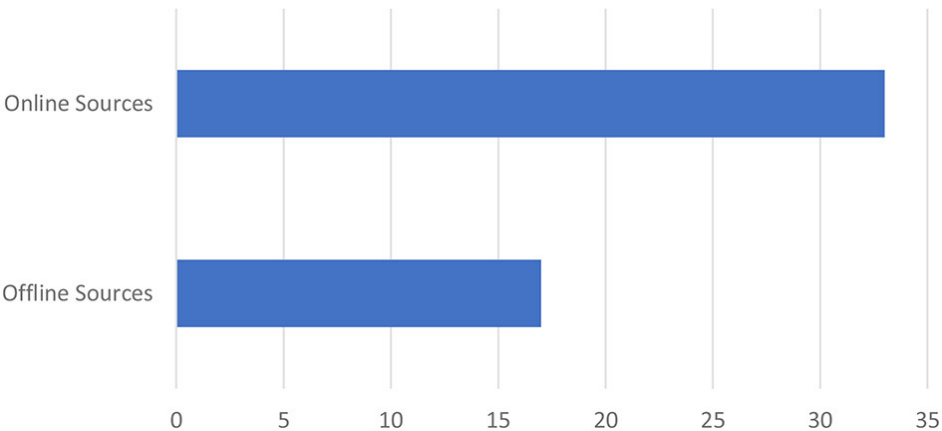
Online vs. offline sources of mis/disinformation.

We identified two primary platforms for the spread of misinformation and disinformation: online platforms (*n* = 33) and offline platforms (*n* = 16). The online platforms included Facebook, Twitter, Messenger, WhatsApp, Instagram, YouTube, websites, Telegram, and Flickr. Among these, WhatsApp (*n* = 9) and Facebook (*n* = 7) were the leading sources of misinformation. The offline platforms included churches, marketplaces, family homes, TV, bars, radio, neighborhoods, and streets, with households (*n* = 5) and neighborhoods (*n* = 4) being the most prominent sources (see Table 1 and Figures 2–4).

**Table 1:** Platforms/sources of mis/disinformation.

**Sources/platforms**	**Freq**
**Offline**	
Churches	2
Market	1
Family homes	5
Tv	2
Bars	0
Radio	2
Neighborhood	4
Street	1
**Online**	
Messenger	1
Twitter	6
WhatsApp	9
Instagram	4
Facebook	7
YouTube	2
Websites	2
Telegram	1
Flickr	1

#### Actors in mis/disinformation

**Figure 5:**
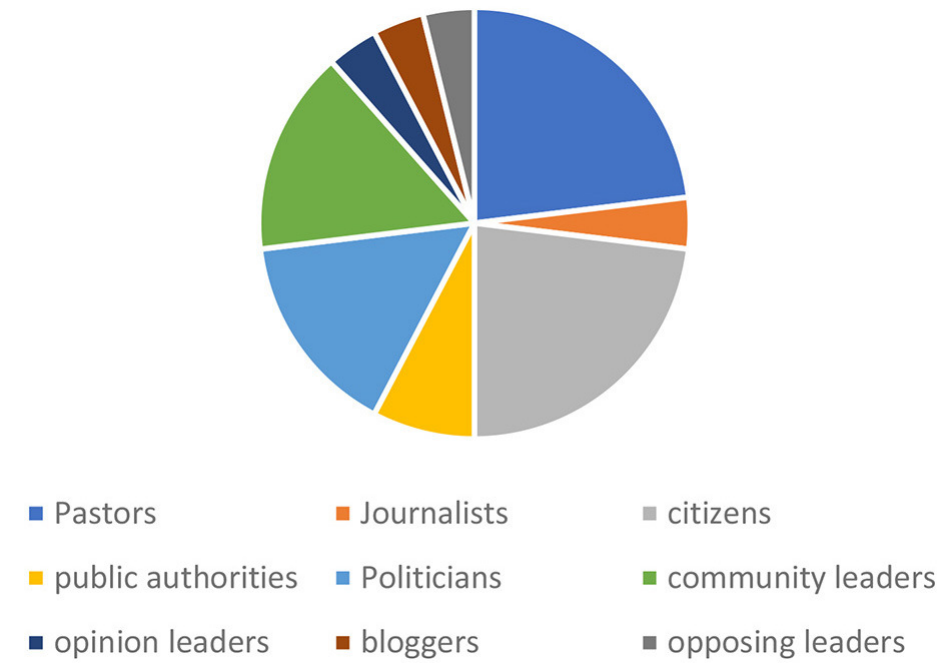
Actors contributions to mis/disinformation.

We identified nine key actors involved in the spread of mis/disinformation. In total, 26 instances of mis/disinformation were documented in the existing literature. Religious leaders, particularly pastors, were responsible for 23.07% of these cases (*n* = 26), while politicians contributed 15.38% (*n* = 26) of the mis/disinformation instances (see Table 2 and Figure 5).

**Table 2:** Actors contributions to the body of evidence of mis/disinformation.

**Actors**	**Freq**
Pastors	6
Journalists	1
Citizens	6
Public authorities	2
Politicians	4
Community leaders	4
Opinion leaders	1
Bloggers	1
Opposing leaders	1
Total	26

#### Types of mis/disinformation

**Figure 6:**
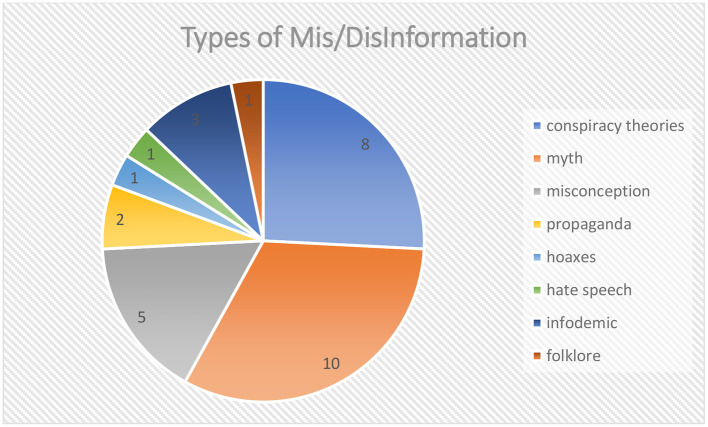
Types of mis/disinformation.

We identified eight types of mis/disinformation, with 31 instances reported in the existing literature. The most frequently reported types were myths (*n* = 10) and conspiracy theories (*n* = 8; see Table 3 and Figure 6).

**Table 3:** Types of mis/disinformation.

**Types**	**Freq**
Conspiracy theories	8
Myth	10
Misconception	5
Propaganda	2
Hoaxes	1
Hate speech	1
Infodemic	3
Folklore	1
Total	31

#### Origin of mis/disinformation

**Figure 7:**
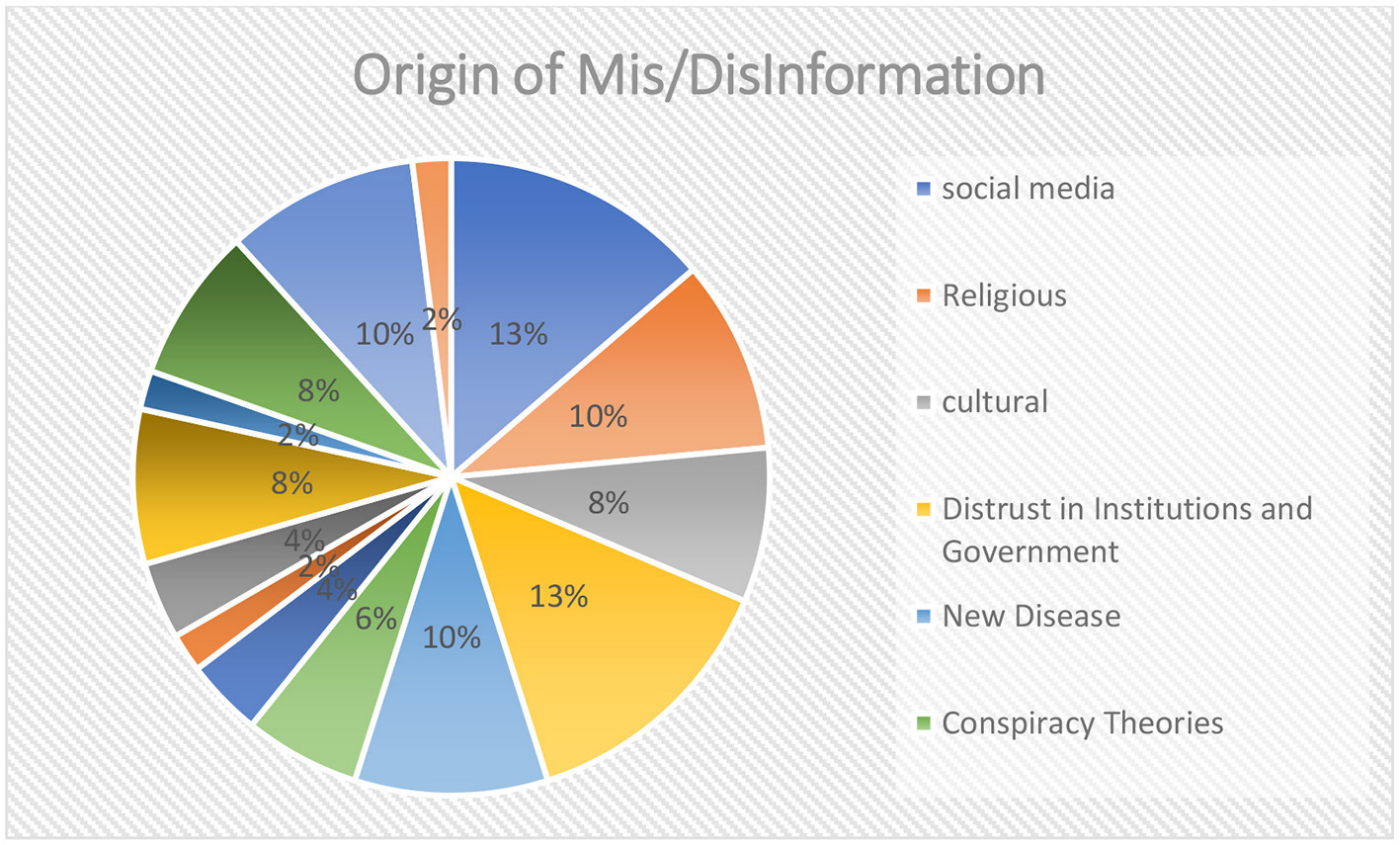
Origin of mis/disinformation.

Six origins of mis/disinformation were identified, with social media, religion, culture, and distrust in government and institutions being the primary sources (see Figure 7).

#### Meta aggregation of disinformation variables

The analysis included studies that reported on the types of mis/disinformation, their sources, the actors involved, specific cases, their impact, and the mitigation strategies employed. Most of the studies reported on the types of mis/disinformation (19 studies), while only a few reported on their impact (nine studies). The most frequently reported variable across all studies was mitigation strategies (112 strategies), followed by cases of misinformation (93 cases). The least reported variable was the number of actors involved (41 actors). This information is summarized in Table 11.

Regarding mis/disinformation on social media, 2,201 original tweets about COVID-19 in Cameroon were identified on X (formally Twitter) between February 2020 and November 2021. These tweets received 1,973,553 likes, 49,724 replies, and were retweeted 142,518 times. The original authors of these tweets had a total of 17,027,578 followers on the platform (see Table 13).

#### Impact of mis/disinformation

The study reported various impacts of mis/disinformation on death (*n* = 2), trust (*n* = 3), non-compliance with government guidelines (*n* = 8), vaccine Hesitancy (*n* = 3), increased spread of disease (*n* = 4), reduced ability of the patient to access health service (*n* = 2), low uptake of research evidence in decision-making (*n* = 1), drugs overdose (*n* = 2), and fear and stigmatization (*n* = 2l; see Table 4).

**Table 4:** Impact of mis/disinformation.

**Impact**	**Freq**
Death	2
Increase distrust	3
Noncompliance with government guidelines	8
Vaccine hesitancy	3
Increase spread of disease	4
Reduces ability of patient to access health service	2
Low uptake of research evidence in decision making	1
Drugs overdose	2
Fear and stigmatization	2
Total	27

#### Exploration of mis/disinformation occurrences: disinfodemic in West and Central Africa

Health misinformation has long been a reality on the African continent and is not unique to the 2020s. Before the COVID-19 outbreak, other novel diseases were similarly plagued by mis/disinformation. These included the HIV/AIDS epidemic in the 1980s, rumors about the polio vaccine in Nigeria in the early 2000s, and Ebola conspiracy theories in the Democratic Republic of the Congo (DRC). The COVID-19 pandemic has seen misinformation spread rapidly across the entire West and Central African region.

Although health misinformation in Africa did not begin with COVID-19, the unique characteristics of misinformation surrounding COVID-19 can be attributed to several factors:

- The emergence of COVID-19 at a time when social media holds significant influence in West and Central Africa.
- The arrival of COVID-19 during a period of widespread conflicts across Africa, which has eroded trust in government and institutions.
- The anxiety and fear stemming from the novelty of the disease.

The impact of social media in fueling the infodemic during the COVID-19 era has been acutely felt across Africa. According to UN Global Pulse, the United Nations Secretary-General's initiative on big data and artificial intelligence, information about COVID-19 has been shared and viewed over 270 billion times online and mentioned almost 40 million times on Twitter and web-based news sites across 47 countries in the WHO African Region. A significant proportion of this information is inaccurate and misleading, and it continues to be shared by social media users, either intentionally or unknowingly, every day (23).

A WHO article published on 30 July 2021 based on data collected in 20 African countries, suggests that false claims about COVID-19 vaccines are among the most widespread myths surrounding the pandemic. The fear of side effects has been identified as the main driver for people's reluctance to become vaccinated (24).

**Figure 8:**
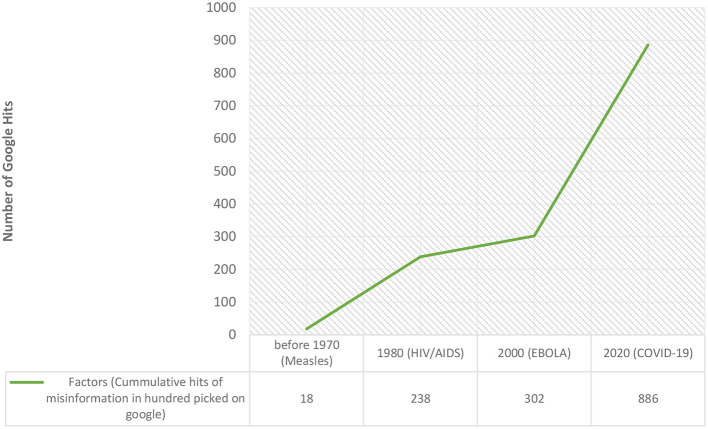
Trends in mis/disinformation with new diseases overtime.

We explored trends in mis/disinformation over time, focusing on diseases such as measles, HIV/AIDS, Ebola, and COVID-19. We conducted a two-decade interval search for existing cases and trends of mis/disinformation in West and Central Africa using Google. Our findings revealed an increase in mis/disinformation over time with each new disease (see Table 5 and Figure 8).

**Table 5:** Factors related to trends in mis/disinformation overtime.

**Measles**	**HIV/AIDS**	**EBOLA**	**COVID-19**
New disease	New disease	New disease	New disease
	Conspiracy theories	Conspiracy Theories	Conspiracy theories
		Conflict	Conflict
			Social media

Four main factors were identified as contributing to this increase over time: the emergence of new diseases, conspiracy theories, conflicts, and the rise of social media.

### Qualitative finding

#### Origin of mis/disinformation

**Figure 9:**
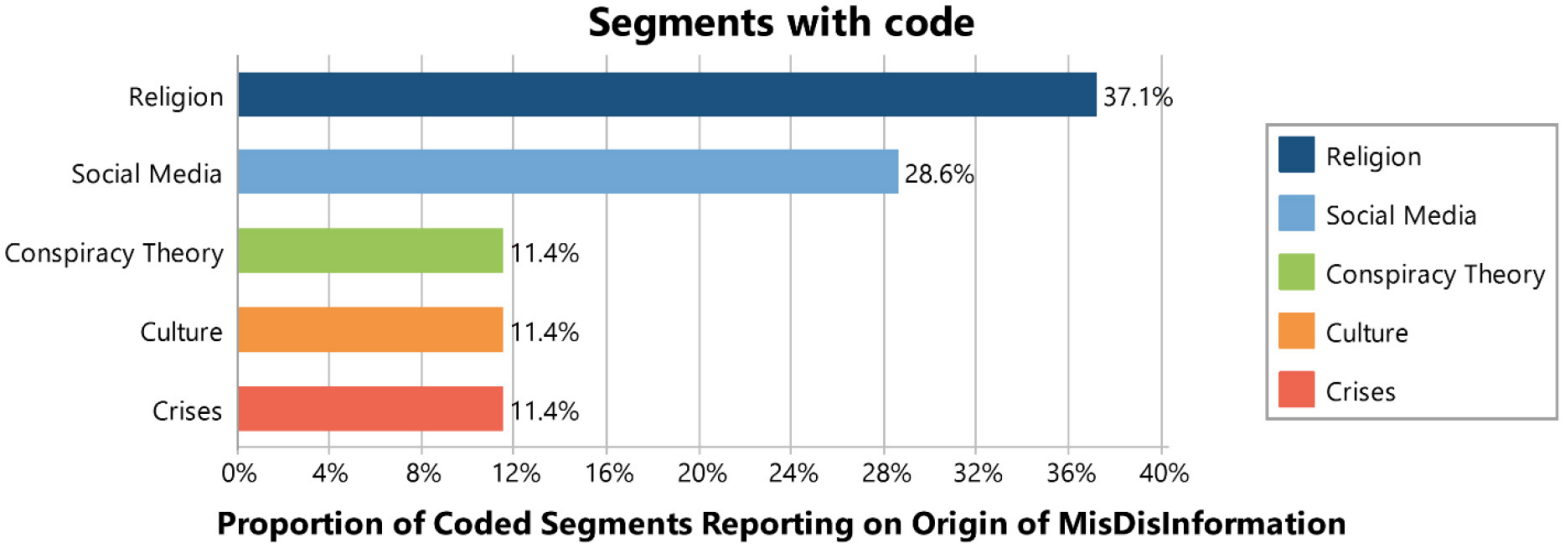
Origin of mis/disinformation.

Our study identified six key origins of misinformation. The distribution of coded segments was as follows: 37.1% for religion (13 out of 35 coded segments), 28.6% for social media, 11.4% for conspiracy theory, 11.4% for culture, and 11.4 for crises (see Table 6 and Figure 9).

**Table 6:** Frequency and percentage of coded segments reporting on origin of mis/disinformation.

**Origin**	**Frequency**	**Percentage**
Religion	13	37.1
Social media	10	28.6
Conspiracy theory	4	11.4
Culture	4	11.4
Crises	4	11.4
TOTAL	35	100.0

#### Cases of mis/disinformation

**Figure 10:**
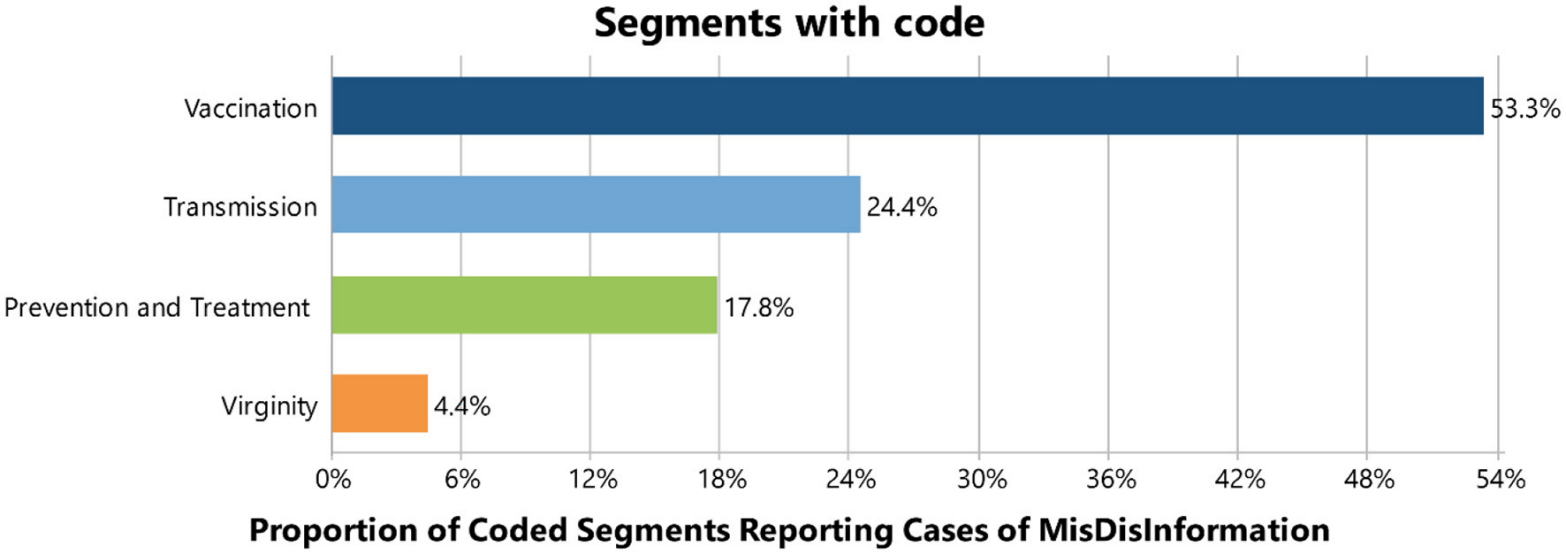
Cases of mis/disinformation.

Our study reported cases of mis/disinformation related to vaccination, disease transmission, prevention and treatment, and virginity. The distribution of coded segments was 53.3% for vaccination, 24.4% for transmission, 17.8% for prevention and treatment, and 4.4% for virginity (see Figure 10).

#### Transmission mechanism of mis/disinformation

The study identified two main platforms through which mechanisms mis/disinformation is spread. The proportion of coded segments was 53.3% for online platforms and 46.7% for offline platforms (see Figure 15).

Online platforms (*n* = 13):

- Facebook (*n* = 5)
- WhatsApp (*n* = 4)
- Websites (*n* = 2)
- Instagram (*n* = 1)
- Google (*n* = 1)

Offline platforms (*n* = 19):

- Public transportation (*n* = 3)
- “Njangi groups” (*n* = 3)
- Churches (*n* = 3)
- Neighborhoods (*n* = 3)
- Traditional media (*n* = 2)
- Workplaces (*n* = 2)
- Markets (*n* = 2)
- Homes (*n* = 1)

**Figure 11:**
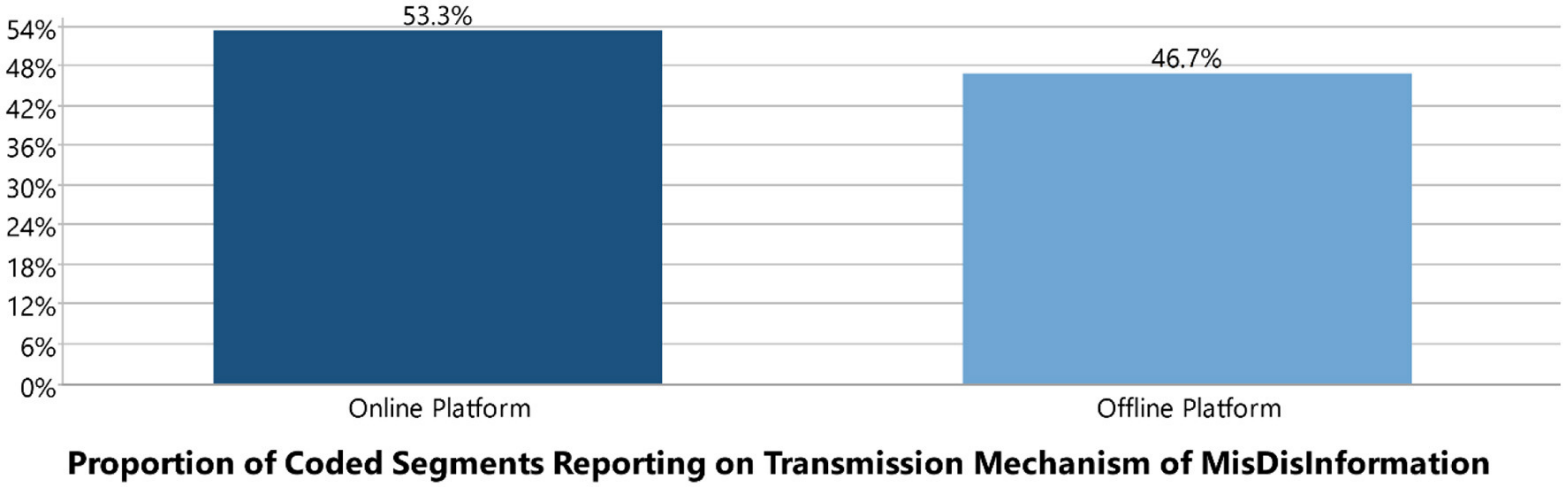
Online and offline platforms as transmission mechanism of mis/disinformation.

**Figure 12:**
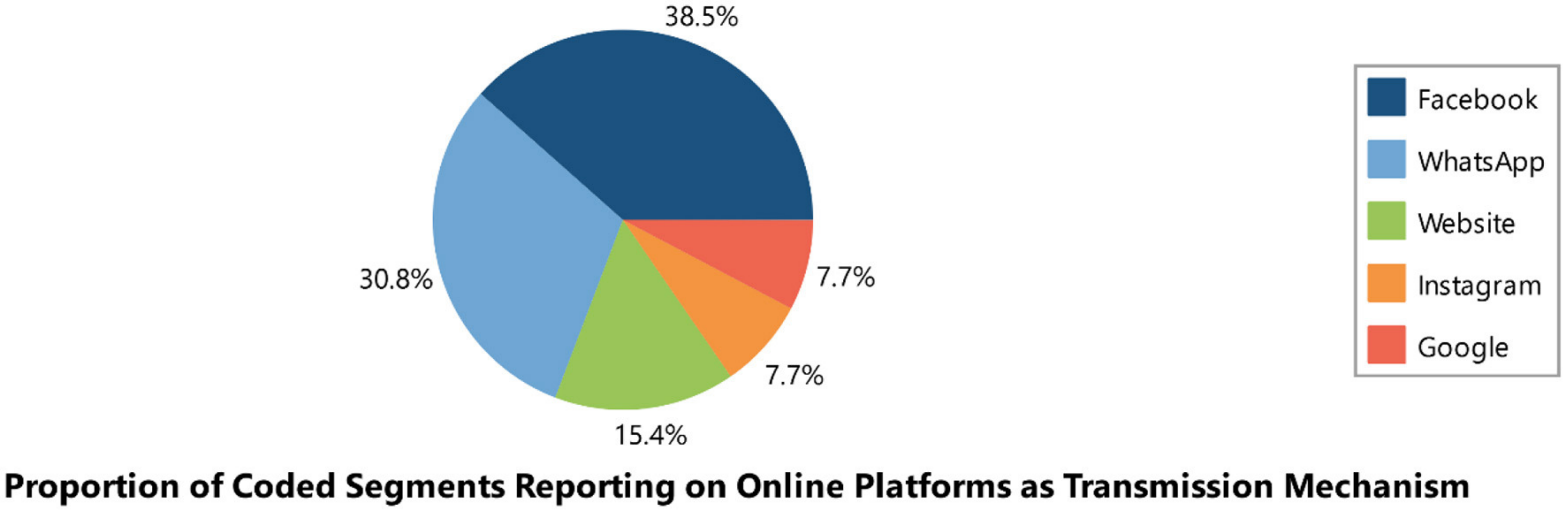
Online platforms as transmission mechanism of mis/disinformation.

**Figure 13:**
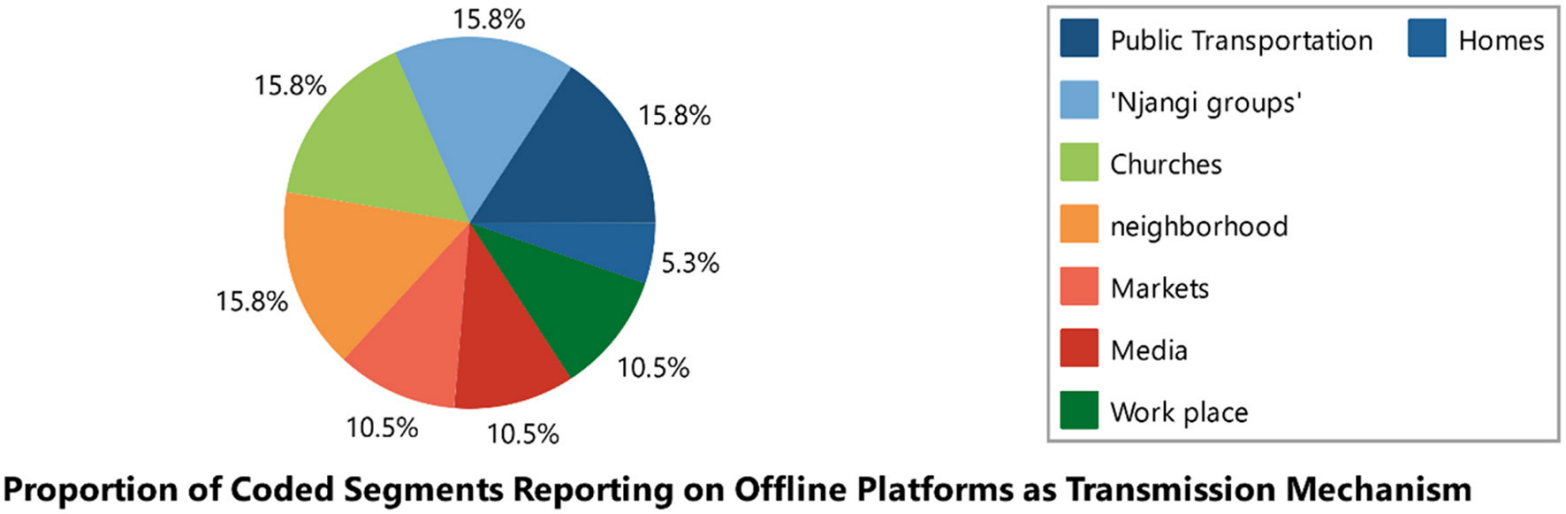
Offline platform as transmission mechanism.

For online platforms, Facebook, WhatsApp, and websites were the leading sources. For offline platforms, public transportation, “njangi groups,” churches, and neighborhoods were the primary sources (see Table 7 and Figures 11–13).

**Table 7:** Frequency of online and offline platforms.

**Platforms**	**Frequency**
**Online platforms**	
Facebook	5
WhatsApp	4
Website	2
Instagram	1
Google	1
Total	13
**Offline platforms**	
Public transportation	3
“Njangi groups”	3
Churches	3
Neighborhood	3
Media	2
Work place	2
Markets	2
Homes	1
Total	19

#### Impact of mis/disinformation

The study reported the impact of misinformation and disinformation on various aspects, including decision-making, vaccine hesitancy, compliance with government policies, and mortality. The distribution of coded segments was as follows: 51.52% (17 out of 33 coded segments) for poor decision-making, 27.27% (nine out of 33 coded segments) for vaccine hesitancy, 15.15% (five out of 33 coded segments) for non-compliance with government policies, and 6.06% (two out of 33 coded segments) for deaths (see Table 8).

**Table 8:** Impact of mis/disinformation.

**Impact**	**Frequency**	**Percentage**
Poor decision making	17	51.52
Vaccine hesitancy	9	27.27
Non-compliance with government policies	5	15.15
Death	2	6.06
Total	33	100.00

The study reported the impact of mis/disinformation on various aspects, including decision-making, vaccine hesitancy, non-compliance with government policies, and mortality. The distribution of coded segments was as follows: 51.52% (17 out of 33 coded segments) for poor decision-making, 27.27% (nine out of 33 coded segments) for vaccine hesitancy, 15.15% (five out of 33 coded segments) for non-compliance with government policies, 6.06% (2 out of 33 coded segments) for mortality (see Table 8).

#### Mitigation strategies of mis/disinformation

**Figure 14:**
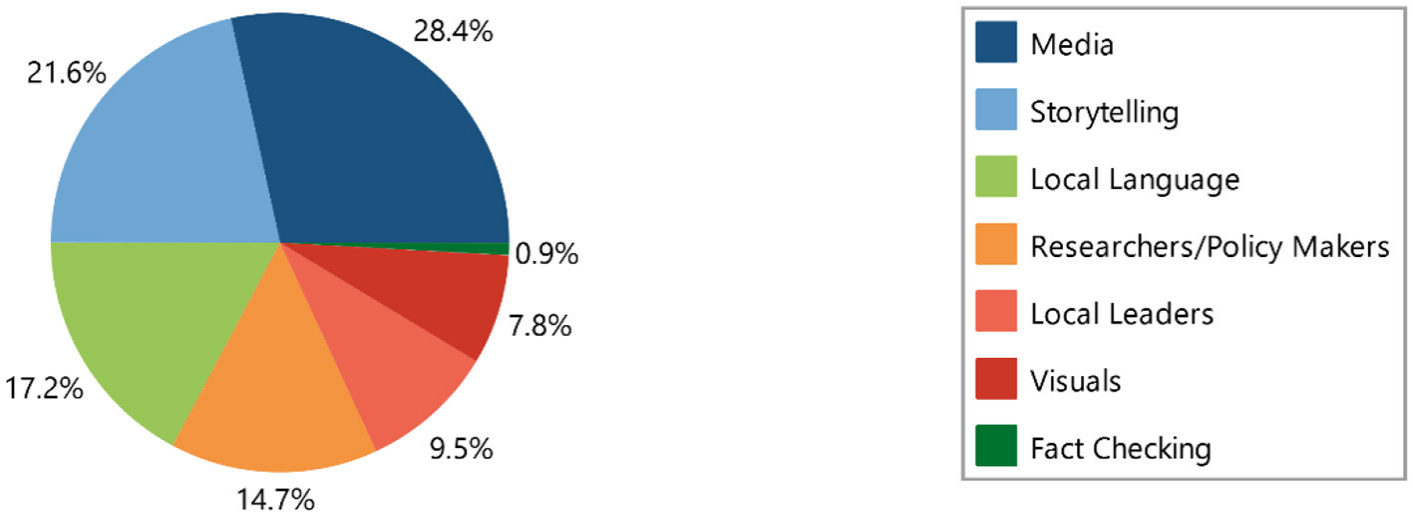
Mitigation strategy of mis/disinformation.

We identified seven mitigation strategies, with a total of 116 coded segments. The distribution of coded segments was as follows:

- Media: 28.4% (33 out of 116 coded segments).
- Storytelling: 21.6% (25 out of 116 coded segments).
- Use of local languages: 17.2% (20 out of 116 coded segments).
- Strengthening relationships between researchers and policymakers: 14.7% (17 out of 116 coded segments).
- Collaboration with local leaders: 9.5% (11 out of 116 coded segments).
- Use of visuals: 7.8% (nine out of 116 coded segments).
- Fact-checking platforms: 0.9% (one out of 116 coded segments; see Tables 8, 9 and Figure 14).

**Table 9:** Frequency and percentage of coded segments reporting on mitigation strategies of mis/disinformation.

**Mitigation strategy**	**Frequency**	**Percentage**
Media	33	28.4
Storytelling	25	21.6
Local language	20	17.2
Researchers/policy makers	17	14.7
Local leaders	11	9.5
Visuals	9	7.8
Fact checking	1	0.9
Total	116	100.00

##### Determinants of success/facilitators of evidence use for EIDM

Facilitators for evidence use in Evidence-Informed Decision-Making (EIDM) were not identified in our study.

##### Strategies to enhance evidence use for EIDM

###### Storytelling

Policymakers should consider using contextually relevant approaches like storytelling to help communicate with the public, as it is a fundamental part of African culture. Storytelling helps the message resonate with the audience, making it easier for the public to remember and share. Informants also recognized storytelling as an effective way to educate children.

When using storytelling for behavior change, it is crucial to consider who tells the story, the audience, the context, and the timing. Storytelling bridges the gap between complex scientific research and the general public, ensuring that even non-literate communities have access to research evidence. Using positive and successful stories to communicate research findings can help limit the spread of misinformation and disinformation.

“*Donc on doit pouvoir calibrer l'information en fonction de chaque cible. Comme je disais, catégoriser la population pour leur donner à toute la population la même information. Une information doit être calibrer dans la population. Il y a plusieurs catégories de population, il y'en a qui sont instruire, il y ‘en a qui ne sont pas, il y a des gens qui sont fortement religieux d'autre qui le sont moins etc. Pour chaque type de catégorie de population vous devez calibrer votre information*.” Male Policy maker Dakar Sénégal: 160–160 (0)

“*…like you group teachers, you teach them some songs in regard to pertinent issues in the society and teachers will in return teach those songs or sing them to their students and those students will take it home. Like for example; like a child of a ‘buyam sellam' when she comes and sing those songs, the buyam sellam will take it to the market and I think it will help circulate the information.”* Female Practitioner Yaoundé Cameroon 3: 27–27 (0)

“*I also think that if the… if the evidence can be presented with the use of songs, storytelling and the use of those things, it's really going to cut across because it is easy to sing a song and while singing that song there are times that you tell yourself that… you unconsciously hum…you sing the song and then you get the meaning of the song.”* Female Practitioner Yaoundé Cameroon 3: 37–37 (0)

“*I think the information first of all has to be clear and simple, yeah and then we use other ways of disseminating information so that the information gets right down to the last person. That's real information, maybe using other strategies like storytelling that has been relegated to the background for a long time now, it's true especially in our setting.”* Male Practitioner Bamenda Cameroon: 94–94 (0)

“*they should be success stories that are told much more than scary stories, stories of people who, who are living better because they are adhering to certain standards and so on.”* Male Practitioner Buea Cameroon: 344–344 (0)

###### Constructive use of the media

A constructive use of both social and traditional media as a mitigation strategy was a key perception shared by the interviewed participants. The public is encouraged to gather information from public broadcast media, as it is the official channel widely used by policymakers to communicate information. On the other hand, policymakers are encouraged to stay updated on the media platforms that are mostly used by citizens, such as Facebook and WhatsApp, to disseminate information effectively.

Researchers are encouraged to share their findings on the same platforms where they collect primary data from the public. The public should also be directed to obtain information from official government websites. Given that citizens are more likely to access information online rather than through traditional print media, policymakers should consider automating information delivery. Social media, being the easiest, most accessible, and fastest way to inform the public, should be utilized effectively. Additionally, policymakers are encouraged to collaborate with telecommunication networks to send messages directly to the public. A TV talk show was organized to support these efforts (see Figure 14).

“*I think that policymakers are supposed to be current; I mean they should be current with the media that is used by the population.”* Female policy maker Buea Cameroon (2): 47–47 (0)

“*So, even though the policy makers, they are skeptical about sending those policies using social media, but that unfortunately is the most accessible method of information to people.”* Female policy maker Buea Cameroon (2): 39–39 (0)

“*People use WhatsApp, people use Facebook groups and those are channels that official sources have not tried to really penetrate. Even government structures or even official organizations that have these platforms, they don't exploit them in a way that the population is open to, so, for me I think it's not just about getting information out there but getting it to where the people you want to receive it are in whatever form they find acceptable.”* Female policy maker Buea Cameroon 1: 75–75 (0)

“*when the time will come we invite the public media, and private media, government owned media and privately owned media because they're all public media because they address the same audience the public.”* Male Policy maker Buea Cameroon: 183–183 (0)

###### Local language

Using the local language of each community to disseminate research evidence can effectively mitigate mis/disinformation. Interviewed participants emphasized that using a language not widely understood by a community can actually lead to misinformation, as the message may need to be understood and recovered. It is crucial to contextualize information to suit the targeted audience and to communicate it promptly before misinformation has a chance to spread. This approach not only limits the spread of misinformation but also enhances evidence-informed decision-making, especially in rural communities.

“*…si je viens m'adresser à une cible et que je te dise un langage que celle-ci ne comprend pas c'est sûr et certain que je fais de la désinformation parce que, il y aura une traduction, une interprétation outre que celle que je donne-moi-même.”* Male Policy maker Dakar Senegal: 41–41 (0)

“*…If you discuss with them in a language that they can understand without quoting all the jargons, without the big names, scientific names and all the things, I think that they could easily understand if it is brought to them locally. So, that is one of the reasons that research evidence is not used. It is not even used at all.”* Female policy maker Buea Cameroon (2): 43–43 (0)

“*…Well, ehmmmm I think that first make this research evidence available in a language that's understood by the population and make it available as soon as it's possible and make it widely available before people have time to twist this information around.”* Female policy maker Buea Cameroon (2): 57–57 (0)

“*…To me the first thing is to put information that everybody will understand talking from the village I'm coming from, I'm from Ndop. I think the language, I think you have to include their languages the languages they speak.”* Female Practitioner Yaoundé Cameroon 3: 37–37 (0)

“*Cameroonians situations you know since we have two languages even the information at times is not well translated. So, it also makes it difficult for those who are part of the English-speaking regions who do not understand French, we also have to understand that texts like that, technical texts if not well translated, it is ehh in short you are getting, you are actually being misinformed. I think that's another.”* Male policy maker Bamenda Cameroon: 61–61 (0)

###### Collaboration between researchers and policymakers

Informants emphasized the need for strong collaboration between policymakers and researchers to enhance evidence-based policymaking. They suggested that the government should allocate sufficient budgets to finance research and collaborate with research institutions, as good decision-making heavily depends on research evidence.

Informants also highlighted the importance of educating policymakers on the value of research evidence in the decision-making process, which could lead to a greater use of evidence in policy decisions. Participants underscored the need for policies that mandate all policy decisions to be evidence-based. On the researchers' side, there is a need for them to provide regular feedback and clear reporting to ensure that policymakers are equipped with the information necessary to make informed decisions.

“*Well, policymakers, to me I believe that policymakers are supposed to work with research institutions, research institutions which should be like universities, should be institutions like IRAD which actually carry out research.”* Male policy maker Bamenda Cameroon: 15–15 (0)

“*For me to take a good decision I must rely on research evidence once that is not there, do not expect that uh decision should be based on evidence. The people need to first buy the idea and know that whatever decision they have to take, they'll need that somebody gives him information about what exists in the field what exists in the whatever domain of human activity they want to take a decision.”* Male Policy maker Buea Cameroon: 223–223 (0)

“*…le chercheur va pouvoir chaque fois allez voir les décideurs, les partenaires au développement; qu'est-ce que je dois faire? est-ce qu'il faut peut-être organiser les réunions pour la distribution de production? … aller voir le pouvoir politique, l'exécutif, le ministère et cetera il va aller voir les députés ceux-là qui vote les lois et cetera.”* Male Policy maker Dakar Senegal: 136–136 (0)

“*…public health that is one challenge we have that activities are carried out, but we don't have an accurate picture of the field because there is poor reporting. So that too could be a challenge for policy makers at higher levels.”* Female policy maker Buea Cameroon 1: 39–39 (0)

“*If you are taking policies concerning agriculture, you should actually know, you should work with ehhh these institutions that are involved that are crosscutting maybe environment, it could be agriculture, and it could other Universities so that information, the policy that they are trying to put should be applicable.”* Male policy maker Bamenda Cameroon: 15–15 (0)

###### Collaboration with local leaders

Community engagement is essential for enhancing the uptake of research evidence in community members' decision-making. Collaborating with community leaders, such as “Njangi” group leaders, church leaders, and traditional authorities, is crucial because they are highly trusted within their communities. This collaboration not only ensures that the information is more readily accepted but also helps these leaders fully understand the message, enabling them to communicate it more effectively to their communities. Working closely with community leaders is also vital for accurately diagnosing the community's needs and challenges.

“*…so that's why the community leaders become important in talking to the people okay? people who are… the persons who are supposed to pass on this information to the public should be the persons that the public can trust.”* Male Practitioner Buea Cameroon: 280–280 (0)

“*secondly also, where people, the government needs to go through things like churches, churches, those are the places where people in fact gatherings. Where people gather, the government especially where people gather on a routine basis”* Female policy maker Buea Cameroon (2): 51–51 (0)

“*I think if people are involved in decision making, if a representative of the population whom people can trust, if they are included in decision making, those decisions will be easily accepted.”* Female policy maker Buea Cameroon (2): 55–55 (0)

###### Visuals

Using various forms of media, such as press outlets, print media, billboards, posters, outlets, and other visual tools, can effectively communicate scientific research evidence to both literate and non-literate citizens. This approach is particularly valuable in remote areas with limited access to television and the Internet, helping to mitigate the spread of misinformation. Participants also emphasized the importance of providing timely updates using visuals.

“*the press media, print media, there should be these big, big posters on the road where they will be talking of COVID.”* Male Practitioner Buea Cameroon: 328–328 (0)

“*…more times in different outlets, print media, hold on media and visual media, print media are like billboards and all that… And then to actually also have some, some of these billboards about the ware of misinformation and… and draw attention to the kind of information that we think is false.”* Male Practitioner Buea Cameroon: 368–368 (0)

“*…people who are in remote areas where there is no connectivity be it radio signals, TV signals, the way to serve them is by print*, you communicate the information to them by posters” Male Policy maker Buea Cameroon: 195–195 (0)

“*…Maybe posters, maybe putting posters there or whatever and changing them regularly on the information they want the public to get yes, it will go a long way to make sure that whatever information is there that they want, people would actually get it.”* Female policy maker Buea Cameroon (2): 51–51 (0)

###### Fact checking

Informants suggested that citizens should be educated on how to verify information they encounter on social media.

“*That's unfortunately a bad thing because people just got introduced to on how to use social media but not how to verify information on social media”* Female policy maker Buea Cameroon (2): 39–39 (0)

“*okay now it will depend on the kind of information, so if we have information that policymakers want to put on social media, they should also be able to tell people how to verify that information.”* Female policy maker Buea Cameroon (2): 53–53 (0).

### Additional findings

Out of the 2,201 tweets analyzed, 50 were selected for data extraction. These tweets reported on the origin, impact, and various cases of COVID-19 misinformation and disinformation. (see Table 10).

**Table 10:** Tweets reporting on origin, impact and various cases of mis/disinformation.

**Main category**	**Sub-categories**	**Number of codes**
Origin	Crises	1
Cases	Cases on transmission	5
	Cases on prevention and treatment	28
	Cases on vaccine	4
Impact	Vaccination	7
	Compliance with COVID guidelines	1
	Death	2

**Figure 15:**
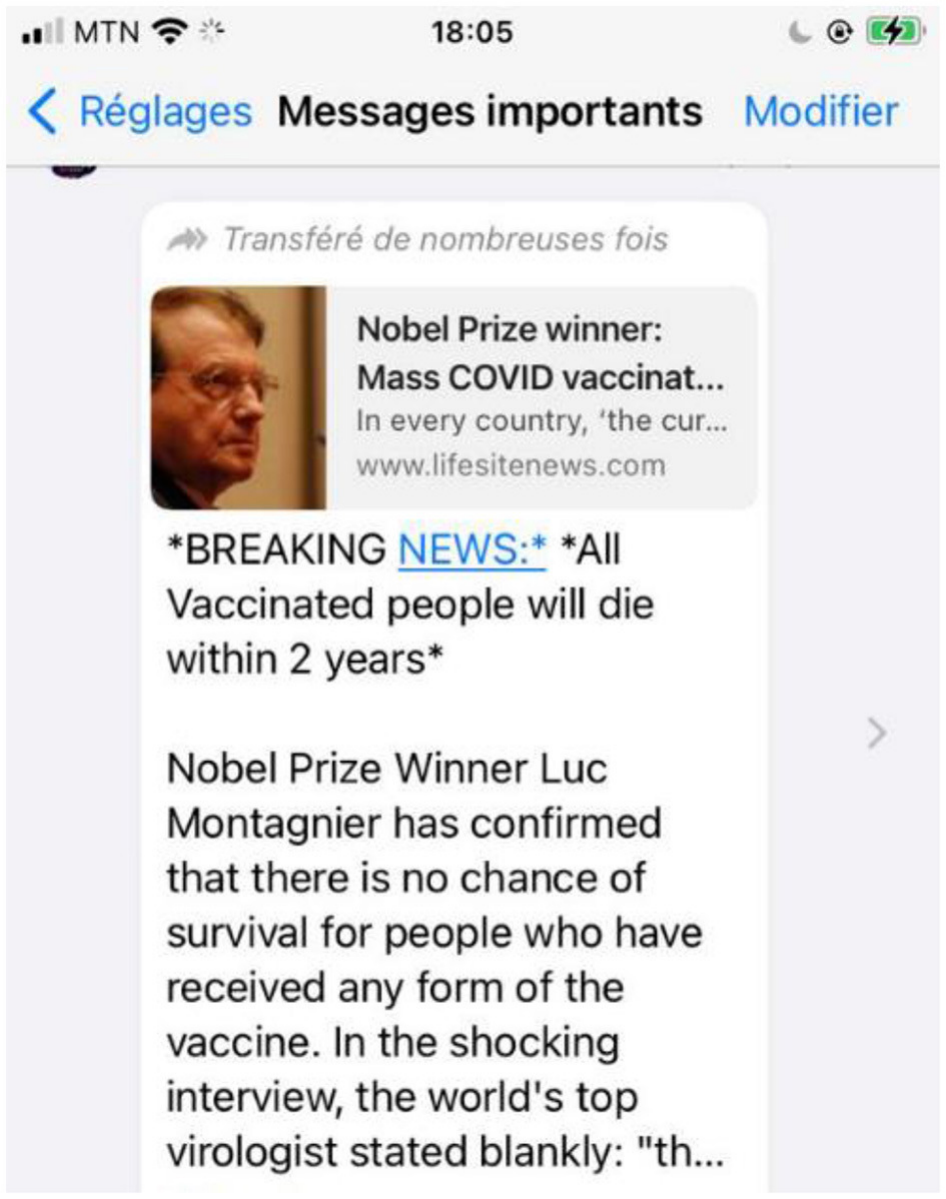
WhatsApp message on misinformation on COVID-19 vaccine.

We collected 141 misinformation stories on WhatsApp, of which 42 were related to COVID-19. These included stories about COVID-19 vaccines (*n* = 25), COVID-19 prevention and treatment (*n* = 11), COVID-19 grants (*n* = 5), and COVID-19 transmission (*n* = 1; see Figure 15).

We conducted an assessment of IDRC-funded projects from 2021 in Senegal, Cameroon, and Nigeria to determine which projects were affected by misinformation and disinformation. A total of 42 program managers were contacted, and a few provided participants for the stakeholder consultation (see Table 11 for results on COVID-19 misinformation in IDRC COVID-19-related projects).

**Table 11:** Synthesis of reported mis/disinformation variables.

	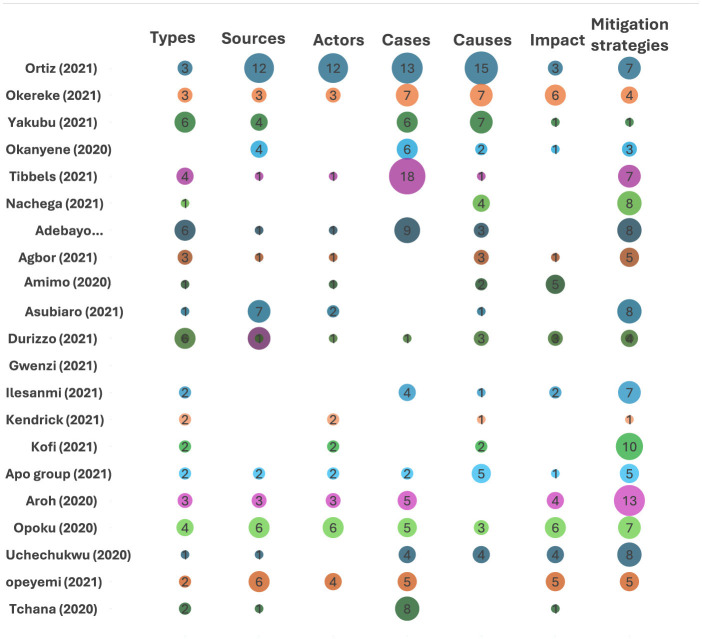

## Discussions

### The theory of disinfodemic

According to Posetti and Kalina (25), the “disinfodemic” refers to the falsehoods that fuel the pandemic and its impacts, exacerbated by the massive “viral load” of potentially deadly disinformation. The UN Secretary-General has described this disinformation as a “poison” and an additional “enemy” to humanity during the COVID-19 crisis. Marshall McLuhan's concept of “information warfare” aptly summarizes this situation, where misinformation during a pandemic can have devastating and deadly repercussions, highlighting the urgent need for significant efforts to mitigate the side effects of this information crisis.

Our study suggests a relationship between mis/disinformation and four key components that contribute to a disinfodemic: *disease novelty (or novelty of a problem/issue), conspiracy theories, conflict, and social media*. This theory is developed based on the most common ways a person in the 21st century encounters mis/disinformation. The rise of mis/disinformation can be associated with the prevalence of social media in the 21st century. The novelty of a disease creates fertile ground for conspiracy theories, which then flourish on social media platforms. Additionally, conflict provides opportunities for these conspiracy theories to gain acceptance, as people often lose trust in governments and global institutions during such times.

During the early days of the COVID-19 pandemic, distrust between nations was high, with world leaders spending valuable time criticizing China instead of focusing on controlling the disease's spread. In preparing for future pandemics, world leaders must consider the role these factors play in influencing the disinfodemic.

### Evidence hesitancy

At eBASE Africa, we define evidence hesitancy as “the failure to accept evidence-based recommendations quickly or immediately, usually due to an underlying reason that may be immediately known or unknown” (26). Evidence hesitancy can be observed among policymakers, practitioners, and citizens alike. In this study, the underlying reasons for evidence hesitancy were as follows:

#### Mis/disinformation

The COVID-19 outbreak has highlighted the significant impact of misinformation and disinformation on the uptake of research evidence. The proliferation of rumors, fake news, and conspiracy theories has engendered considerable fear and mistrust concerning the effectiveness of COVID-19 vaccines in Africa, resulting in widespread vaccine hesitancy. This misinformation not only influences individual vaccination decisions but also affects public policy decisions regarding the implementation of vaccination programs. Insights from interviewed participants further illustrate these challenges:

“*I for one, I was really… I don't think I was really interested in the COVID vaccine…… yes I wasn't interested with the COVID vaccine because, I've watched videos of people eh eh taking the vaccine and falling, having strange reactions so I wasn't interested.”* Female Practitioner Yaoundé Cameroon 3: 13–13 (0)

“*Il y a beaucoup d'informations qui circule dans cela donc vous voyez chacun, il y'en a plein vous avez vu au Nigeria ce qui s'était passé par rapport à la vaccination pendant longtemps on a interdit les milliers des 1000000 de personnes à se vacciner.”* Male Policy maker Dakar Senegal: 140–140 (0)

#### Crises

The African context is often characterized by persistent conflict, which breeds mistrust in government institutions. This mistrust, in turn, limits the uptake of research evidence by citizens. This study highlighted the impact of such crises on vaccine uptake, showing that mistrust in government has been a significant factor leading to vaccine hesitancy.

“*And besides we are in this part of the world, this part of the country, the North West and the South West where there is crisis already, an ongoing crisis and the people see every strategy that the government is making as something against them.”* Female policy maker Buea Cameroon (2): 13–13 (0)

#### Culture

African culture holds a central place in the lives of its people. Consequently, when research evidence conflicts with cultural beliefs, there is a strong likelihood that individuals will reject the intervention in order to preserve their cultural identity.

“*…those traditional persons, who believe that they are also protected by their ancestors, who have a certain belief that everything else which does not conform to the way in which they have brought up is just false information… the traditional person said that no, it is the way they have being living their life from the beginning, they cannot turn around and ask them not to live their lives the way they're living the god of their land will not allow it happen.”* Male Practitioner Buea Cameroon: 113–113 (0)

#### Religion

Religion can also contribute to evidence hesitancy, particularly when religious teachings contradict scientific research evidence. For example, in our study, informants reported experiences related to COVID-19 and family planning where religious messages conflicted with scientific recommendations. Some participants recounted instances where religious leaders portrayed the COVID-19 vaccine as the “mark of the beast,” suggested that relying on the vaccine indicated a lack of faith, or emphasized that prayer should take precedence over medical interventions. Additionally, there were claims that family-planning vaccines lead to both promiscuity and infertility among girls. These beliefs have contributed to evidence hesitancy among church members, which, in turn, eventually affects the public policy decision-making process.

“*d'autres dissent que la planification familiale pervertir leurs femme nos filles”* Male Policy maker Dakar Senegal: 140–140 (0)

“*et des religieux qui sont contre là planification familiale n'est ce pas il y a d'autres qui donne les informations sur la vaccination qui disent que la vaccination c'est simplement pour amener nos filles a ne plus faire d'enfants donc que c'est pour stériliser non filles”* Male Policy maker Dakar Senegal: 140–140 (0)

“*bon si on parle de santé de la reproduction en générale il y'a certains religieux ou biens des prédicateurs qui sont le plus souvent contre la planification familiale, ces dirigeants qui restent sur leur position et qui disent qu'ils ont raison sur tout et qui font une autre interprétation baser sur d'autre considération religieuse qui ne sont pas toujours celles que nous avons donc ces gens-là peuvent considérablement saper le travail que nous faisons.”* Male Policy maker Dakar Senegal: 104–104 (0)

“*when you confront the pastor that God has given us knowledge that we should be able to use it to solve our problems he maybe interprets it as being having less faith, not having faith.”* Male Practitioner Buea Cameroon: 192–192 (0)

#### Social media

Our study identified a relationship between social media and evidence hesitancy. With the outbreak of COVID-19, there has been a significant increase in misinformation and disinformation circulating on social media platforms. These falsehoods are disseminated through various formats, including videos, memes, images, and text, reaching a wide audience. Such misinformation creates doubts in the minds of social media users, contributing to evidence hesitancy.

“*my mom told me not to get vaccinated because there is a video that she was sharing that she has seen a video of so and so happening and if I get vaccinated it was going to harm me”* Female policy maker Buea Cameroon 1: 69–69 (0)

### Stakeholders' experiences with evidence use

Stakeholders identified opportunities for enhancing the use of evidence, although in many cases, the use of evidence during the COVID-19 pandemic was hindered by the prevalence of mis/disinformation. Given that policymakers, practitioners, and citizens often access mis/disinformation through more digestible formats, such as social media, rumor networks, and so on, research evidence faces significant competition.

**Figure 16:**
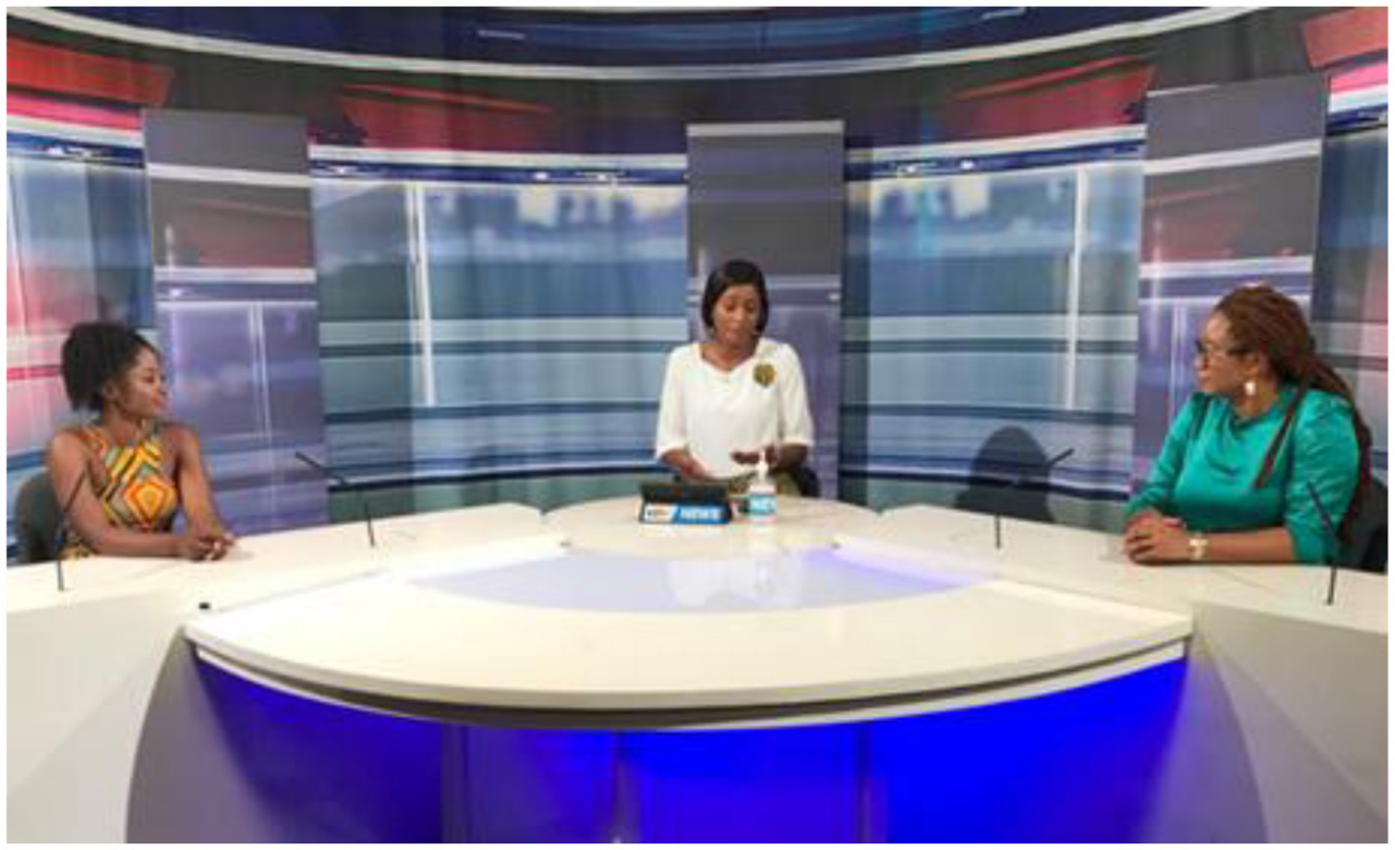
eBASE Africa using the National Media in Cameroon to create Awareness on the danger of infodemic in an era of pandemic [World Evidence-Based Halthcare (EBHC) Day].

Knowledge brokering could play a key role in bridging this gap by leveraging existing social media platforms to reach citizens effectively. Knowledge brokering should exploit existing social media platforms and broadcast media especially for citizens (See Figure 16). Knowledge brokering should also use evidence portals, policy briefs, and evidence summaries to reach stakeholders. It is also essential for research initiatives to consider the co-creation or co-production of knowledge resources with and for stakeholders to promote ownership and facilitate the use of evidence (27). Furthermore, considerations for disability should be integrated into these processes (28).

### Digitalization: a present opportunity

Given the high penetration rate of mobile devices in rural Africa, mobile phone technology presents a significant opportunity that has yet to be fully exploited. Mobile phones have the potential to overcome literacy barriers and can even help initiate and improve literacy. The use of voice, images, graphics, geolocation, and videos offers countless possibilities to empower populations in remote communities. Despite this potential, policymakers, developers, and citizens have not yet fully capitalized on this opportunity. Digitalization, data innovation, and artificial intelligence should be recognized as essential components of sustainable development goals and research (29–31).

### Prioritizing strategies for evidence use

Our study identified the relationship between policymakers and researchers as the most effective strategy for increasing the uptake of research evidence by policymakers. Strengthening this relationship can help reduce the impact of misinformation and disinformation on public policy decision-making.

For citizens, stakeholders prioritized storytelling, use of local language, collaboration with local leaders, and visuals to mitigate the effects of misinformation on the use of research evidence in West and Central Africa.

### Equitable evidence use

Closing the equity evidence gap largely depends on contextual considerations, such as how accessible and comprehensive the research evidence is. For research evidence to achieve more equitable outcomes, it must be made available in a manner that supports innovative and improved decision-making for all (32). However, within the African context, equity in evidence remains a challenge. This is often due to the complexity of research terminology and its need for more contextual relevance.

Our study revealed that the low uptake of research evidence is primarily due to its exclusionary nature. Informants emphasized that for research evidence to be made accessible to all, considerations of language, literacy rates, and cultural aspects must be integrated into the production and communication of the research evidence.

### Cultural considerations in evidence use

The wide research practice gap between research practices and their application in Africa highlights the need for more inclusive strategies to ensure the research evidence is well understood by policymakers and the broader community, and effectively implemented. For evidence-informed decision-making (EIDM) to be successful, research evidence must be culturally sensitive. Cultural sensitivity includes attributes such as knowledge, consideration, understanding, respect, and tailoring to ensure effective communication, effective intervention, and satisfaction (33). Our study identified the use of local languages and storytelling as key cultural considerations for EIDM.

To fulfill the principle of making research evidence available to all, it is essential to integrate African culture into the production and communication of evidence (34). Policymakers, researchers, and practitioners must have an in-depth knowledge of the culture of each beneficiary community, respect that culture, and tailor interventions to reflect it.

Our study highlighted local language as a crucial component of African culture and an effective tool for communicating research evidence. Africa is one of the most linguistically diverse continents, with thousands of spoken languages (35). Additionally, the African continent also has a high rate of illiteracy, with the majority of the population living in rural communities (36). Research evidence is often produced in official languages such as English and French, which are not widely spoken or understood by much of the African population. To bridge this gap, it is important to communicate research evidence in the local language of each beneficiary community. The spread of mis/disinformation has been closely linked to ineffective communication.

“*Il y a une cible au niveau communautaire qui n'est pas alphabétisée. Si je viens leur parler de données probantes, c'est sûr et certain des gens qui ne comprendront absolument rien à ce langage.”* Male Policy maker Dakar Senegal: 41–41 (0)

“*Sorry to me the first thing is to put information that everybody will understand, talking from the village I'm coming from I'm from Ndop. I think the language, I think you have to include their languages the languages they speak.”* Female Practitioner Yaoundé Cameroon 3: 37–37 (0)

The African tradition of storytelling remains one of the continent's oldest and most enduring forms of communication. It has long served as a means to pass on traditions, convey codes of behavior, and maintain social order. However, important stakeholders, such as non-literate communities, which constitute a significant portion of the African population, are often left out of the evidence ecosystem. Therefore, it is important to consider storytelling as a tool for brokering and translating research evidence to these non-literate communities (37). This approach can help reduce the spread of mis/disinformation, enhancing the use of research evidence, particularly among those who are not scientifically literate.

Interviewed participants emphasized that making research evidence more accessible requires the use of storytelling, not only because it is deeply rooted in African culture but also because it effectively communicates messages to a broader audience.

“*I think the information first of all has to be clear and simple, yeah and then we use other ways of disseminating information so that the information gets right down to the last person. That's real information, maybe using other strategies like storytelling that has been relegated to the background for a long time now, it's true especially in our setting.”* Male Practitioner Bamenda Cameroon: 94–94 (0)

“*I think when you introduce the storytelling issue I think that, it just came to my mind that that was the best way you talk to teens, to younger children and it is the same way I think we can talk to even elders”* Female policy maker Buea Cameroon (2): 43–43 (0)

In March 2020, shortly after the outbreak of COVID 19, the popular African artist Koffi Olomide released a song titled “Corona Virus Assassin” to raise awareness about preventive measures. This song garnered 2.9 million views, with ~29,000 likes and over 3,000 comments on YouTube alone, highlighting the power of storytelling in communicating scientific research evidence. In the song, Olomide emphasizes the importance of adhering to COVID-19 preventive measures such as staying indoors, practicing social distancing, and washing hands.

A few months later, in May 2020, Cobhams Asuquo released a song titled “We Go Win (Corona),” which received over 39,000 views on YouTube alone. Asuquo's song not only raised awareness about COVID-19 preventive measures but also addressed the dangers of fake news, urging the public not to share misinformation. The song was performed in Pidgin, a local language widely spoken by non-literate communities in the English-speaking parts of Africa.

…*No shaking hands with your neighbor*

*Blow them a kiss from afar*

*Use soap and water to wage war…*

…*Self-isolate for the sake of…*

*All the people wey you love ooo*

*Don't go around spreading rumors*

*Cos fake news won't help anyone*

### Institutionalization of EIDM

According to the definition adopted by Li et al. (38), institutionalization involves “…developing accepted norms and rules and sustaining effective working relationships between relevant policymakers and research institutions.” These norms and rules encompass concepts such as transparency, accountability, citizen engagement, openness, deliberation, and contestability, all of which contribute to enhancing the quality and credibility of evidence-informed-priority-setting and decision-making. As Conaway (39) noted, “And if the goal is to drive research use among policy makers, then an obvious first step is to put policy makers and researchers in the same room.”

Our study identified the strengthening of relationships between policymakers and researchers as a crucial step toward institutionalizing research evidence for EIPM. Policymakers are encouraged to network with research institutions to better use research evidence in policy decision-making. Our study highlighted that the ineffectiveness of EIPM often stems from a lack of collaboration between researchers and policymakers. An effective collaboration would result in consistent feedback and reporting from researchers and increased availability of research evidence for policymakers throughout the policy process (40).

This study also highlighted the rise in the spread of mis/disinformation with the rapid increase in social media users in Cameroon. Such mis/disinformation can greatly dilute the fruits of research and funding efforts (see Table 12). There is a notable similarity between the spread of mis/disinformation and the spread of epidemic diseases (41, 42).

**Table 12:** IDRC investment in projects from 2021 in Nigeria, Cameroon and Senegal for mis/disinformation.

**Sector**	**Country**	**Study**	**Funding (CAD)**
COVD-19 pandemic	Cameroon	Mbaye (2024)	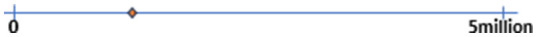
		Kong (2023)	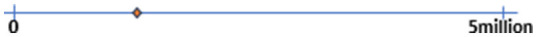
		Alban (Active)	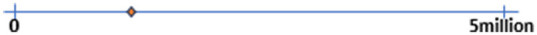
	Nigeria	Stroebel (Active)	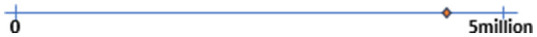
		Agyepong et al. (2022)	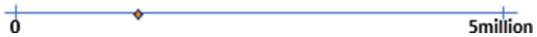
		Ndung'u (Active)	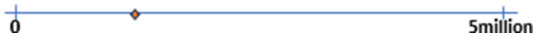
		Shipalana (Active)	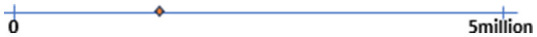
		Dossou (2023)	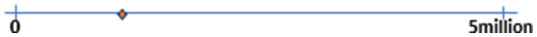
		Gillwald (Active)	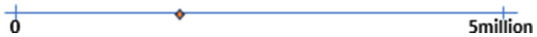
		Mariara (2023)	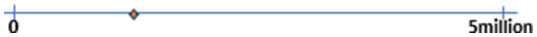
	Senegal	Faye (2024)	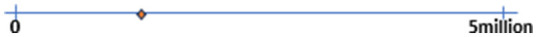
		Marchand (2022)	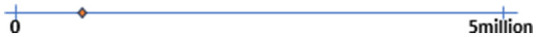
		Vandeplas (2022)	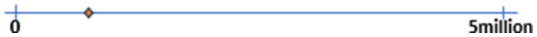
		Reed (2022)	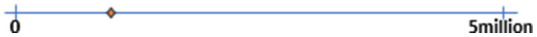
		Hathie (2022)	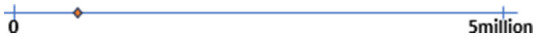
		TRAORE (2021)	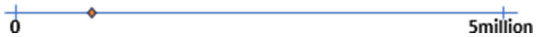
		Maiga (2021)	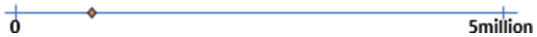
Ebola virus disease	Senegal	Kobinger (Active)	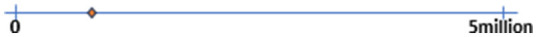
Advocacy against early marriage and sexual violence/sexual and reproductive health	Nigeria	Pambe et al. (2022)	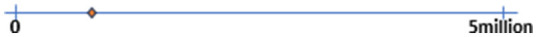
		Chimaraoke et al. (2023)	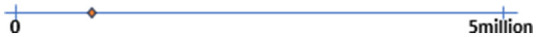
		Mbachu (2023)	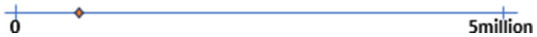
		Alemika (2022)	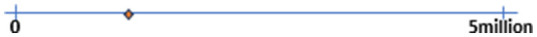
		Chimaraoke et al. (2023)	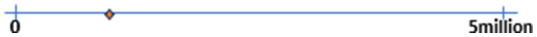
	Senegal	Moreau (Active)	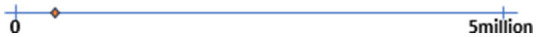
		Sall (Active)	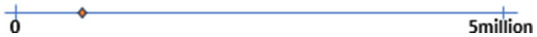
		NDIAYE et al. (Active)	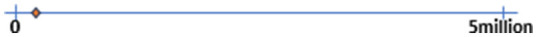
		DIADHIOU (Active)	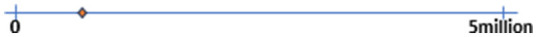
		SALL (2021)	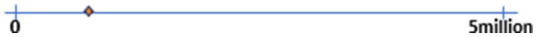
		Traore (2023)	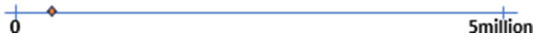
		IDRC (Active)	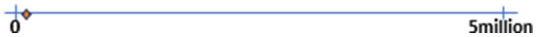
		IDRC (Active)	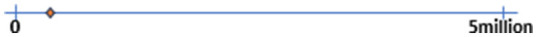
		Ataguba (2024)	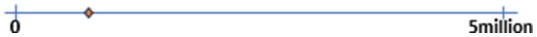
		Faye (Active)	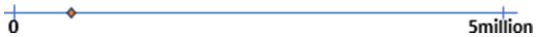
		Faye (Active)	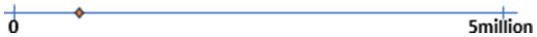
Social entrepreneurship	Cameroon	Foretia (2023)	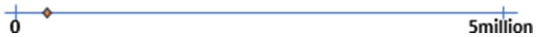
Tobacco control	Nigeria	Onyekwena (2021)	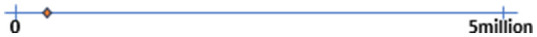
	Senegal	Walbeek (2023)	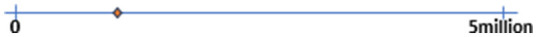

The Susceptible-Infected (SI) model, which describes the interaction between susceptible (S) and infected (I) individuals during pandemics such as COVID-19, can be adapted to model the effect of mis/disinformation. Suppose *I* represents the share of the population that has been mis/disinformed, and *S* represents the share of the population that has not yet been mis/disinformed. By considering the characteristics of the authors of such mis/disinformation, such as their profession, number of followers, the number of people they follow, and the number of likes and retweets their posts generate (see Table 13), we can estimate the rate of flow of mis/disinformation from the population in I to that in S. If this rate is significantly high, it will dampen the impact of research and funding efforts.

**Table 13:** IDRC funded projects and mis/disinformation tweets about COVID-19 in Cameroon not limited to X (formerly Twitter) users' resident in Cameroon.

Number of original misinformation tweets about COVID-19	2201
Total number of retweets	142,518
Total number of replies	49,724
Total number of likes	1,973,553
Total number of followers of original authors	17,027,578

## Data Availability

The original contributions presented in the study are included in the article/supplementary material, further inquiries can be directed to the corresponding author.
